# Histopathology of nasal mucosa and inflammatory changes in nasal wash of symptomatic rhinitis patients

**DOI:** 10.1186/1939-4551-8-S1-A208

**Published:** 2015-04-08

**Authors:** Loreni Kovalhuk, Nelson Rosario Filho, Monica Lima, José Ederaldo Telles

**Affiliations:** 1 Federal University of Parana, Brazil

## Background

The extent of epithelial damage in allergic (AR) and nonallergic rhinitis (NAR) and its association to inflammatory changes in nasal washes (NW) are not fully understood.

## Objective

Investigate the relationship of inflammatory cells in NW and the level of epithelial damage and basement membrane thickening (BMT) of the upper airway mucosa

## Methods

Total nasal symptom score (TNSS), NW and turbinate biopsy specimens were obtained from 36 AR and 20 NAR patients. Atopic patients had positive skin prick test to *D.pteronyssinus* (35/97%) and *L.perenne* (18/50%) extracts from Hollister-Stier. Total and differential cell counts were evaluated by a quantitative method of nasal cytology; albumin and IL-8 concentrations were determined in the supernatant of NW. The epithelium damage and BMT were assessed on H&E-stained sections by staging system. Statistical analysis was performed by nonparametric Mann-Whitney U test for comparison between cell counts and differences in frequencies by Fisher exact test.

## Results

The median age was 24.5ys. (14-58). TNSS was higher in AR (9 [1-18]) as compared to NAR (6.5 [0-12]) (p=0.01). Total cell and neutrophil counts, as well as albumin and IL-8 levels were not different in NW of AR and NAR patients. Median eosinophil count in nasal fluid (ECNF) was higher in AR (3% [0-66]) than in NAR (1% [0-21]) (p<0.01). ROC curve analysis and AUC for ECNF accuracy in distinguishing AR from NAR, has showed a cut-off value of 4%, AUC=0.71. ECNF was ≥4% in 44% of AR and 20% of NAR patients; at this point the probability of atopy was 80%, with 44% sensitivity and 90% specificity. Epithelial damage was more frequent in AR (94%) than in NAR (65%) (p<0,01). According to the presence of BMT, NW of AR patients without BMT had higher median eosinophil (3% [1-7]) and neutrophils (47.5% [0-87]) counts compared to eosinophils (1% [0-4]) and neutrophils (12% [0-23]) counts in NAR. On the other hand, in the presence of BMT, there were no differences in the NW of AR and NAR patients. The intragroup analysis showed that neutrophil count was higher in NW of NAR patients with BMT (45% [12-83]) than without BMT (12% [0-23]) (p<0.01).

## Conclusions

The best cut-off value of ECNF to discriminate atopic patients was 4%. Despite of differences in mechanisms of inflammatory reactions in rhinitis, airway remodeling assessed by BMT is associated with similar cellularity in NW of both AR and NAR.

